# Three Cases of Tubercular Peritonitis Operated on by Abdominal Section during the a Year1Read before Toronto Medical Society.

**Published:** 1891-01

**Authors:** James F. W. Ross

**Affiliations:** Lecturer on Abdominal Surgery, Toronto University, and on Gynecology, Woman’s Medical College, Toronto; Gynecologist to Toronto General Hospital, St. Johns’ Hospital for Women, and Toronto Dispensary; Corner Wellesley and Sherborne Streets


Buffalo Medical and Surgical Journal

Vol. XXX. JANUARY, 1891. No. 6.

### Original Communications.

### THREE CASES OF TUBERCULAR PERITONITIS OPERATED ON BY ABDOMINAL SECTION DURING THE YEAR.[1]

By JAMES F. W. ROSS, M. D.,

Lecturer on Abdominal Surgery, Toronto University, and on Gynecology, Woman's Medical College, Toronto; Gynecologist to Toronto General Hospital, St. Johns' Hospital for Women, and Toronto Dispensary.

I thought that perhaps the histories of the three following cases might be of general interest to the members of this prosperous society, a society composed largely of members who fill the honorable calling of general practitioners. We all meet face to face a deadly foe — tubercle — a terrible foe, no matter in which organ or in what limb he assails us, a foe that nearly always is the victor, and we the vanquished. It may be that during our lifetime, in the light of recent researches, the tables may be turned, and this dreaded enemy may become as inert as the meanest of the parasites in the presence of an efficient parasiticide.

Case I.[2] Mrs. K., *aet.* thirty-six, referred to me by my father, Dr. James Ross, Sr., III. para, youngest seven years; no miscarriages; was last unwell nine months ago. Was quite regular previous to that time. Abdomen began to enlarge about two months ago. She is now subject to cough and shortness of breath. Has pain in abdomen resembling the sticking of knives; this shoots up and down the abdomen, but is more severe on the right side. She has never been jaundiced. The enlargement has been rapid, and seemed to her greater on the right than on the left side. She has the face peculiar to patients with ovarian dropsy.

On examination I found the abdomen much enlarged. Greatest girth thirty-seven inches. From umbilicus to crest of right ilium, 8¼

1. Read before Toronto Medical Society.

2. Where the present tense is used, the extracts from my notes of the cases taken at the time are left unaltered.

inches. From umbilicus to crest of left ilium, $8\frac{3}{4}$ inches. On palpitation a hard mass was to be felt about $1\frac{1}{2}$ inches above the level of the umbilicus, but to the right of the median line. On percussion, bowel resonance was obtained down the linea alba, and in the flank, but it was a muffled resonance. On sitting up, there was evidence of the presence of ascitic fluid, though this was not definitely obtained when turned on either side. Fluctuation could be made out, but with a feeling of thickened walls intervening between the fingers. The test made by voluntary elevation of the head, and consequent contraction of the recti-muscles over the surface of the tumor, was satisfactory ; a bulging took place in each flank with the ready displacement of the fluid free in the abdomen. Uterus was found to be empty.

*Diagnosis.*— I felt sure that there was a thickening of the peritoneum, and free fluid within the abdomen. Whether malignant or tubercular, I did not venture to say.

*Treatment.*— Advised exploratory incision. I saw the patient again, but failed to detect solidification of any part of either lung. The sputum was not examined. Operation performed March 28, 1890. Several of the active staff of the Woman's Hospital were present. I found the abdomen filled with serous fluid ; no flocculi. The peritoneum was about $\frac{1}{4}$ of an inch thick, and studded with tubercular nodules. Above the hard mass to be felt externally, and mentioned above, was found to be produced by a thickened omentum, glued by thickened tissue to the abdominal wall, forming a conglomerate mass. The bowels were glued together in the upper abdomen and back in each loin. No tubercles were seen studding the parts of the bowels visible, but they were much reddened and inflamed. Tubercles might have been seen upon a more minute inspection, but they were not so readily visible as they were in a case to be related further on. Ovaries and uterus were quite normal and free from adhesions. The abdomen was washed out with a forcible stream of hot water, and drained through a glass drainage-tube placed in the pouch of Douglas. Before operation the temperature had been ranging between 99° and 100°. Pulse from 80° to 90°. On the day of operation, March 28th, at 5 P. M., temperature, 101.6°, pulse, 90° ; at 9.30 P. M., temperature, 98.6°, pulse, 90°. On the 29th, temperature reached 99.2° ; on the 30th, 99° ; on the 31st, 100.8° ; on the 2d April, 102.4° ; on the 4th and 5th, 102.4. The temperature remained elevated until she left the hospital. The drainage-tube was taken out and a rubber one inserted. The wound healed readily. Stitches were taken out on the seventh day. The drainage-tube was shortened day by day, and nothing but a very small opening remained when the patient left the hospital some weeks after. During the latter part of her stay, symptoms of deposit of tubercle in the apex of the right lung could be readily made out. Cough became more troublesome, and sputum was like that of a phthisical patient. Tubular breathing, increased vocal fremitus and tactile fremitus, could be obtained. An offensive diarrhea began to trouble her on the third day after the operation, and continued at irregular intervals until she went home. The hardness of the lump present in the abdomen before operation seemed to melt away in a marvelous manner. The abdomen did not refill while under my care.

After leaving the hospital she slept with a window open near her bed and contracted pleuro-pneumonia. I saw her once and advised that she be sent to her to the general hospital under one of the physicians, with the promise that I would call in to see her occasionally while attending to my patients in the gynecological department. We could not readmit her to the Woman's Hospital on account of the annoyance arising from her troublesome cough.

She did not follow the advice given, and I have not seen her since. On inquiry I find she died from tubercular disease of the lungs two months ago, ten months after the onset of disease. Whether there was any return of the abdominal symptoms I could not ascertain. The lung affection was, however, the most prominent.

Case II. M. B., *aet.* sixteen, single. Unwell only three times in her life. Now suffers from amenorrhea. I saw her first on the 16th June, 1890. She was then thin and emaciated, and looked very ill; temperature, 101°, pulse, 110. She said that about February, 1890, she noticed that her abdomen was enlarging. She was put upon a course of treatment and the swelling seemed to reduce. A month ago she again increased in size. She complains of pain in her sides, *i. e.*, in her loins. No edema is present. The uterine fundus cannot be felt. No ovaries to be found by bimanual examination. The sound could be passed but a very short distance. The abdomen was found very much enlarged. On palpation no definite tumor could be made out, but I found again between the fluctuating waves a peculiar muffled sensation experienced in the former case, and with my recent experience I felt sure that here again was a thickened peritoneum. Irregular tympanitic notes were elicited over the abdomen.

Her family history showed that her mother died of pyloric stenosis, supposed to have been malignant. Father still living and well. Father's sister died of tuberculosis. No other information could be obtained. Bowels move two or three times daily. Tongue is much coated at its posterior part.

*Diagnosis.* — In this case I made a positive diagnosis to the class, and said that the case was one of tubercular peritonitis. There were all the symptoms of free fluid in a thickened peritoneum, and the more definite symptoms of ovarian tumor, or other tumor, were absent.

Advised operation.

Operation performed June 18, 1890. On cutting down found peritoneum much thickened and studded with tubercular nodules, looking here and there like little teats. The intestines were matted together, pressed backward into each loin, and upward by the fluid, and looked angry and inflamed. No broad ligament was present. There were no folds of pelvic peritoneum. The pelvic cavity ended in a round cul-de-sac, at the bottom of which sat an ill-developed cervix uteri. No fundus uteri could be definitely made out, and no ovaries or tubes could be felt.

Dr. A. H. Wright, who assisted me with the operation, passed his finger into the pelvis, and could not make out any positive fundus uteri, nor ovaries nor tubes. If the uterus was complete, it was very tiny. About three quarts of fluid were drained away, and the cavity was well flushed with hot water. A glass drainage-tube was used, and a rubber one was substituted for it later on. The rubber one was gradually shortened, and finally left out altogether. The wound healed by first intention. The temperature fell on the day after the operation from $102\frac{1}{6}°$ to 98°, and never rose above 100° afterwards. The patient went home on the 31st day of July, 1890, in perfect health. I have written for her history since her return. By the kindness of Dr. McKibbon, her physician, the following particulars have been obtained, dated November 27, 1890: "I saw your patient to-day. A small opening remains in the tract of the drainage tube, surrounded by granulations, and continues to discharge, slightly, a thin yellowish fluid. She has enjoyed comparatively good health since her return. Appetite is extremely good. Suffers no pain. All the functions are normally performed, with the exception of the bowels. She suffers from constipation. The lungs are entirely free from disease. I can notice a slight enlargement in the left lumbar region, (I noticed this myself before she left the hospital,) and the whole abdomen presents a prominence that did not exist on September 15th, but the hard, rigid feeling of the abdomen then noticed has, to a great extent, disappeared. If there is any dropsical effusion, it is small in amount, and cannot be discerned. She has improved in flesh since the operation, but her condition is not to be compared with what it was prior to the commencement of her trouble last January."

Case III. H. P., *aet.* thirty-two. Married ten years, III. para; youngest two years; no miscarriages; menses began at fourteen years; always quite normal until last Winter when the discharge became more profuse and sometimes lasted for two weeks. No excessive pains. Was always able to go about. In appearance she is somewhat pale, with a coppery tinge; looks like a person suffering from a cachexia, anemic. Her chief pain is referred to the neck of the bladder. Micturition very frequent. No chest symptoms.

*Urine.*— Acid, pale amber, contains mucus, pus, a few red blood corpuscles, squamous epithelium and crystals of oxalate of lime. Tests for albumin and sugar not recorded by my clerk, She says that three or four weeks ago she had a fever with chills and sweats. These chills and subsequent perspirations came on every evening for three or four days. She had severe pains in the pelvic region. She was supposed to be suffering from kidney disease. Appetite good, no vomiting. Bowels regular and move once or twice a day.

Has been in poor health for two years, ever since the birth of her last child. She has not been confined to bed until recently. On examination, under an anesthetic, I made out very distinctly a mass on each side of the uterus, and had no hesitation in pronouncing the case to be one of double pyosalpinx.

Operation advised and performed on the 16th of August, 1890, in the presence of several of my colleagues and students. The abdomen was not enlarged, and felt quite normal. On opening it the omentum was found to be adherent. The visceral and parietal peritoneum was studded with tubercle and somewhat thickened. The intestines were matted together. No fluid was present in the abdominal cavity. The inflammatory action had evidently been of the dry, adhesive variety. With some little difficulty I separated the adhesions and found both Fallopian tubes distended by fluid, and with their walls very much thickened. They were not removed, owing to the general condition found. I drew out a coil of intestine to show the students its condition. It was so studded with the tubercular deposit, that one could scarcely find a clear space as large as a ten cent piece. The mesentery was also very little affected. There was very little fat present. The bowels had the same appearance as that found in the preceding cases, namely, a red, inflamed appearance. It was impossible to wash out this case, owing to the adhesions of the bowels. A drainage-tube was left in for three days. The temperature never rose after the operation above 99.4°.

The patient went home. An unfavorable prognosis was given to the husband. In answer to a letter of inquiry I received the following from him a few days ago : December 1, 1890. "My wife is not well. For about three weeks after her return she was about the same as when she left Toronto. She then seemed to improve for two weeks and was going around the house. Then just before noon weakness came on. She is not, however, confined to bed. Our boy broke his arm six weeks ago, and she seemed to catch cold while looking after him. She has coughed a good deal since, chiefly at night time. When quiet she does not suffer much pain, but after moving about she has great pain in the neck of the bladder, and gets no relief until she has passed water. At times the urine is very dark colored and bloody, and when left to stand it settles in the dish like jelly. Appetite, until a week ago, has been good. It is now, however, very fair, but nothing she can eat seems to give her strength. She has chills and fever at bed time, and then about four o'clock in the morning she sweats. Her pulse is from 100° to 120°."

From this there can be no doubt as to the tubercular nature of the trouble. Kidney and bladder, as well as intestines and Fallopian tubes, are, no doubt, affected.

This concludes the histories of these cases. They have been given somewhat exhaustively and may have tried your patience. But forming, as these cases do, such a puzzle when they occur in adults, it is desirable that the symptoms should be accurately recorded.

In connection with these cases I wish to mention another operated upon by a strange coincidence on the 28th of March, 1890, the very day upon which I opened the abdomen of Case No. I. The patient was sent to the woman's hospital under my care with a temperature of 103° and a pulse of 120, presenting all the signs of a sub-acute peritonitis. The abdomen was filled with fluid and extremely sensitive, and the patient was moaning with pain. On opening the abdomen I found it filled with serous fluid. Intestines were much distended with flatus. The peritoneum was studded with nodules and teats exactly like those found in the case just operated on half an hour before (Case No. I). A suppurating ovarian cyst was found on the left side and a suppurating hematocele was distending the right broad ligament. The tarry fluid characteristic of hematocele, mixed with pus, came out when the trocar was plunged into the mass. Here was what appeared to me to be a sub-acute peritonitis caused by the condition of the pelvis (a condition presumably not of tubercular origin) with a peritoneum presenting to the unaided eye the exact appearance of a tubercular peritoneum. Certainly a curious experience for one day. This woman had unfortunately waited too long and died twelve hours after an operation done, when she was so weak that she nearly died upon the table. Owing to the death of the patient we have none of the after symptoms found, in two of the three cases related, to guide us in determining the tubercular or non-tubercular nature of the disease.

I have seen in the practice of others several cases of tubercular peritonitis.

From my three cases we can learn several things.

All of the patients appeared to be very ill. The temperature was elevated in each case. Abdominal pains were present in each case—in one, in the abdomen like knives sticking into her, in another chiefly in the sides of the abdomen, and in the third case the pains were chiefly pelvic. As regards menstruation, in Case I. she did not menstruate for nine months; in Case II. amenorrhea for many months, but only unwell three times in her life, due, no doubt, to the imperfect development of the internal genital organs. In Case III. we have an increased menstrual flow. In the two cases in which fluid distended the abdomen amenorrhea was present. In two cases we had a collection of fluid accompanying the disease, while in one case the condition produced an adhesive or dry peritonitis. The ages varied — sixteen years, thirty-two years, thirty-six years. Two cases were multiparous women; the other a single young girl. In Case No. I. the enlargement was noticed only two months, in Case No. II. for three or four months, and in No. III. the disease had been in progress about two years, though no enlargement of the abdomen was noticed. Enlargement, therefore, was rapid. In Case I. the internal genitals were found to be healthy, in Case II. they were not developed, and in Case III. they were diseased. In each case the bowels were adherent to one another, were congested in appearance, and in Case III. were thickly studded with tubercle. The omentum was much thickened in Case I., was not much affected in Case II., and was adherent to the abdominal wall and studded with nodules in Case III. In two cases, I. and II., the peritoneum was very much thickened, while, though thickened in III., not to such an extent.

The question might arise: Was No. II. a case of tubercular peritonitis, or simply a case of sub-acute peritonitis, such as that found in the case of a suppurating ovarian cyst, mentioned above? That a sub-acute inflammation will produce just such nodules is, I think, proven by the case related, unless it was also of tubercular origin. But in Case II. there was no history of injury and no evidence of any abdominal affection that would have been likely to have originated a sub-acute inflammation. We have, therefore, to lean to the belief that it was of tubercular origin, for want of something more definite.

The fact that the patient has remained well for a period of five months, does not prove the curability of tubercular peritonitis. Nor is this proved by the recovery of the patient. We must be able to say, without a shadow of a doubt, "such and such nodules were tubercular and not inflammatory." Several writers claim that they have found the bacillus tuberculosis in cases that have recovered subsequent to operation.

There can be no doubt that tubercle is, as Roberts says, the most common and important new growth connected with the peritoneum. Fagge and Bristowe have both classified many cases. One of the most valuable papers on the subject is that of Fenwick. He classifies forty-seven cases found in the records of the London Hospital. He divides tubercular peritonitis into acute and chronic; and the chronic into adhesive, ascitic, suppurating.

Roberts's division is:

1. General acute tuberculosis, of which tubercular peritonitis forms an unimportant part, in which the peritoneum is studded with miliary tubercle.

2. Secondary to tubercular ulcers in the intestines.

3. As an independent disease that may be associated with phthisis, but is usually primary and generally occurs in young persons. Osler, in following Spillman in his classification, leaves out pelvi-peritoneal tuberculosis and peritoneo-pleural tuberculosis, and I think rightly so, and simply accepts:

1. Acute miliary tuberculosis (given by Roberts).

2. Chronic caseous and ulcerating tuberculosis (found in the purulent division of Fenwick).

3. Chronic fibro-tuberculosis (the adhesive form of Fenwick).

We now come to look for a moment at the *morbid anatomy* from which these classifications have been made.

I will give a short synopsis of the forty-seven cases of Fenwick, and it will be very similar to any synopsis given of any number of cases.

*Peritoneum.*—Thickened in twelve cases. In one case it was one inch thick; in one case, three-quarters of an inch thick.

*Omentum.*—The chief cause of the formation of a hard tumor. Thickened in eight cases.

*Liver Capsule.*—Frequently thickened. In one case as hard as cartilage.

*Spleen Capsule.*—Frequently thickened, and parts in neighborhood frequently matted together.

*Mesenteric Glands.*—Rarely enlarged sufficiently to form tumors.

*Liver.*—Frequently cirrhotic. In twenty out of 165 cases this was found, and there was a history of intemperance in every case but one.

*Spleen.*—Frequently studded with tubercle and sometimes studded with abscess cavities.

*Kidneys.*—Frequently studded with tubercles, also found granular, and one case pelves were dilated and the kidneys cystic.

*Bladder.*—Found with tubercular deposits and also found ulcerated.

*Generative Organs.*—Affected in nine cases out of sixteen. This is not a satisfactory statement, because the original classification was not accurately made. In one place the uterus and appendages were taken together, and then in another Fallopian tubes were taken alone. Fenwick thinks that this, perhaps, accounts for the difference existing between his statement and that of other authors in this connection.

Fagge says that the Fallopian tubes are nearly always affected. Moxon, that the disease affects chiefly the outer ends of the tubes showing that the disease spreads into them from the peritoneum.

Fagge says that the epididymis or testes are often affected in the male. He apparently thought that the establishment of puberty predisposed to the implication of the tubes, because he says that out of eight cases in women, all but one in whom puberty had been established, had coëxistent tubal disease. Osler gives 1-4 with tubes affected; Immermann, 7-16 with tubes affected.

*Lungs.*—Fagge found them affected in 7-9 cases; Bristowe found them affected in 42-48 cases; Fenwick found them affected in 33-38 cases.

*Caseation* was found between intestinal coils in three cases; of the abdominal parietes, one case; cretaceous deposit in one case.

*Ascitic Fluid.*—Fenwick found in thirteen cases out of forty-seven; in two cases it was bloody serum. This is evidently found much more frequently in malignant diseases of the peritoneum; in three cases it was pus. Pus is found much more frequently in children.

*Sex.*—Fenwick gives sixteen females out of forty-six cases; Osler gives 131 females out of 191 cases; Bristowe, Fagge and Fenwick all give a preponderance of males. Osler thinks the recent laparatomy records are more to be relied on, and uses them. But this can hardly be accepted. Many cases of the disease die in obscurity if they have no ascitic collection to necessitate operation. The cases operated on are brought more prominently forward and are more frequently recorded.

*Age.*—Fenwick says the largest number affected are from fourteen to twenty years of age.

Osler says from twenty to forty years.

*Family History.*—Many cases show no hereditary predisposition.

*Previous Health.*—Fenwick says six out of forty-seven suffered from cough previous to the outbreak of the disease, but in no case was preëxisting advanced phthisis found.

*Onset of the Disease.*—In some cases very sudden, having to go to bed at once suffering from pain and tenderness over the abdomen and distention. Only one case lay with the knees drawn up. In some, symptoms were like those of gastric ulcer—pain and vomiting; in others like typhoid fever, having even the rose colored spots.

In some a hardness was found in the right iliac region simulating disease of the cecum; some cases had no pain; some cases began with an obstinate diarrhea, or vomiting and diarrhea; temperature was usually elevated; pulse somewhat quickened.

*Menstruation* in some was increased; in some, stopped for many months before the first outbreak.

*Duration.*—One case died in one month; four cases died in two months; one case died in six months; one case died in seven months. They had been well previously. It is, however, difficult to say definitely when the disease begins.

Pigmentation of the skin is noticed in some cases (also in my Case III.), and is supposed to be due to tubercular disease of the supra renal capsules.

From what I have already said, the main symptoms in typical cases will vary according to the nature of the case.

Differential diagnosis will have to be made from many diseases. In children the condition can be readily guessed at. "They look old; have shrunken features; a tumid belly," says Fenwick; "their limbs are wasted; they suffer pain; they are tender on pressure, and have obstinate diarrhea." In young girls, however, the diagnosis is not always easy. It is only a few weeks since I saw a case simulating closely my second case just related. The chief point was, however, the sudden onset of the symptoms. I could not account for them and for their severity, unless they were the result of the strangulation of the pedicle of an ovarian tumor. The girl looked in the face just as M. B. did. Her abdomen had just such an indefinite feeling of a thickened peritoneum. She had severe pain and evident peritonitis. She had been in this condition for some time. At the operation we found a gangrenous ovarian tumor with soft friable edematous walls and a black pedicle just about twisted off. Fluid was free in the peritoneal cavity, and the peritoneum was thickened and very much congested. Such, then, is very likely to be mistaken for tubercular peritonitis.

Such semi-solid tumors as those produced by thickened omentum and agglutinated bowels can readily be mistaken for peritoneal cancer.

Cases of the adhesive variety, as Case III., cannot be accurately diagnosticated unless some affection of the chest coëxists to give us the clue, unless we are led by some pelvic symptom to open the abdomen. The main points that led me to my diagnosis in Case II. were: the irregular tympanitic sounds, something out of the common; the presence of a fluid with lax walls surrounding it; the indistinct wave of fluctuation, giving one the idea of a thickened intervening wall, a totally different feeling to that produced by a wave through a collection of fluid in the sac of an ovarian cyst, a distinct but a far-away wave, not like the feeling produced by colloid contents; the peculiar feeling of the abdominal wall when grasped in the hand; the flattening of the tumor by the tension of the recti; the high temperature and coated tongue; the cachectic and emaciated appearance, rapid pulse, and the fact that I had recently had Case I. to guide me. Now, in closing, I would like to say a few words regarding treatment. Tapping the peritoneum is exactly analogous to tapping the pleura. We cure pleurisy, so often tubercular, in this way; then, why not peritonitis? If we wait long enough in many cases of pleurisy, nature performs the cure, and so in the abdomen nature does the same. In either case absorption takes place and adhesions are formed. Many cases of complete absorption of fluid from the peritoneum in cases of tubercular peritonitis are on record — cases in which the post-mortem examination has verified the previous existence of tubercular lesion. Tubercular disease in the lung often becomes innocuous. Then, why not in the abdomen? Some held that to cure these cases we must open and wash out the abdomen. Then cases were cured without washing or the use of medicaments. Then the simple aspiration of the fluid was found sufficient. But we have records showing that cases have, so to speak, cured themselves. Does operation hasten the cure? One thing is certain, and that is, that many operated on die of subsequent phthisis, just as they do if left alone. From the statistics of Maurange forty-one per cent. were doing well one year after operation. I believe that exploratory incision should be made in every such case. It clears up the diagnosis and prolongs life. I believe that neither washing out nor drainage are necessary. The best treatment is incision, or, in other words, a paracentesis abdominis done without using that dangerous instrument, the trocar. With the knife and the finger the operator knows where he is going and what he finds.

Corner Wellesley and Sherborne Streets.

